# Virus inactivation in stored human urine, sludge and animal manure under typical conditions of storage or mesophilic anaerobic digestion†

**DOI:** 10.1039/c6ew00311g

**Published:** 2017-03-17

**Authors:** Loïc Decrey, Tamar Kohn

**Affiliations:** Laboratory of Environmental Chemistry, School of Architecture, Civil and Environmental Engineering (ENAC), École Polytechnique Fédérale de Lausanne (EPFL), CH-1015 Lausanne, Switzerland

## Abstract

Viruses represent major disease transmitting agents carried by human excreta and animal manure. Understanding virus inactivation is therefore essential in preventing microbial spread due to inadequate treatment of these materials. Here, we investigated the inactivation kinetics of the single-stranded (ss) RNA phage MS2, DNA phages T4 and ΦX174, andthe double-stranded DNA human adenovirus in stored human urine, sludge, and animal manure, at temperatures and pH valuestypical of storage under naturally occurring conditions or mesophilic anaerobic digestion (<40 °C). The ssRNA phage MS2 was most readily inactivated in all samples compared to the other viruses tested. This is consistent with previous findings in wellcontrolled buffer solutions of similar composition, where inactivation was found to be governedby bases (NH_3_, carbonate, hydroxide) that catalyze the transesterification and cleavage of the ssRNA. Correspondingly, MS2 inactivation kinetics in real matrices could be adequately modelled by only taking into account the effects of temperature, pH, carbonate and ammonia on the integrity of ssRNA. DNA viruses were more persistent compared to MS2;however, inactivation in selected sludge and manure samples proceeded at faster rates compared to well-controlled buffersolutions of similar composition. This indicates a contribution of microbial or enzymatic activity to inactivation of DNA viruses. Overall, this study identifies the most important factors contributing to inactivation of viruses in human excreta and manure, and highlights the differences in inactivation kinetics and mechanisms between ssRNA and DNA viruses.

Water impact

Viruses are among the most resistant pathogens in human excreta and manure (HEAM). A better understanding of the kinetics and mechanisms driving virus inactivation in HEAM is instrumental to design and optimize HEAM treatment and reduce pathogen dissemination into the environment. In particular, it enables to evaluate virus stability, identify resistant viruses, and assess the public health risks posed by virus transmitted by HEAM.

## 1 Introduction

Prior to reuse or discharge, human excreta and animal manure (HEAM) need to be correctly managed to avoid the transmission of pollutants (macro- and micro-pollutants) and pathogens to the environment.^1–4^ If adequately treated, however, environmental pollution and public health issues resulting from the disposal of HEAM can be minimized.^5–8^ Organized excreta containment, collection and treatment is one of the pillars in breaking the transmission cycle of fecalorally transmitted pathogens.^9^ Currently only a small percentage of human excreta collected is safely managed, in particular in cities in lower-income countries, though on-site storage or treatment offer an opportunity to reduce thepathogen load before disposal.^10,11^ On-site treatment should in particular focus on viruses and helminths, since they were shown to be the mostpersistent pathogens in excreta, in particular during storage at ambient temperature.^12–14^ Their inactivation kinetics, and the factors that promote inactivation in HEAM, however, remain poorly understood.

In previous work, we characterized virus inactivation in well-controlled, homogenous laboratory solutions under conditions of pH, temperature and chemical composition typically encountered during storage or mesophilic anaerobic digestionaof HEAM.^15,16^ Single-stranded (ss)RNA viruses, such as enteroviruses or MS2 coliphage, were shown to be rapidly inactivated. Inactivation resulted from base-catalyzed transesterification of the ssRNA, which causes the genome to cleave.^15^ The efficiency of a base to induce inactivation depends on the p*K*_a_ of its conjugated acid and its concentration. Under the solution conditions considered in Decrey *et al.*,^15^ the most important bases promoting inactivation were hydroxide and ammonia, though other bases such as (bi-)carbonate also contributed. Based on this mechanistic insight, a model to estimate the inactivation rate constant under typical urine, sludge and manure storage conditions was established for MS2 coliphage, which was shown to be a conservative surrogate of other ssRNA viruses in such conditions.^16^ Using the solution composition as the input, this model was able to accurately estimate the MS2 inactivation rate constant at 35 °C and over a pH range of 7.5–9.5. In contrast to ssRNA viruses, double-stranded (ds) RNA and both ssDNA and dsDNA viruses, which are not amenable to genome transesterification, exhibited low inactivation rates under the same conditions. More extreme conditions of pH or temperature, such as those encountered in thermophilic digestion or alkaline treatment, were required to achieve appreciable inactivation rates.^16^

Compared to laboratory solutions, real HEAM matrices exhibit a higher level of complexity: they contain particles, additional chemical constituents (*e.g*., metals or organic acids), as well as live communities of microorganisms that may contribute to virus inactivation. The current study extends our understanding of virus inactivation fromlaboratory solutions to real matrices associated with on-site HEAM storage (sludge, stored human urine, animal manure). Specifically, we aim to determine if the principles of virus inactivation established in well-controlled solution also apply to HEAM matrices. Our objectives were i) to validate the previous approach to estimate MS2 inactivation for the more complex conditions encountered in HEAM; and ii) to establish if the inactivation trends observed in laboratory solutions for viruses with different genome types correspond to those in real matrices. To attain these objectives, the pH, temperature and ion composition of different (diluted) stored urine, sludge and manure solutions was determined, and MS2 inactivation in these solutions was monitored and compared to the predicted inactivation. Furthermore, the inactivation kinetics of the base-sensitive ssRNA phage MS2 as well as the more resistant dsDNA phage T4, ssDNA phage ΦX174 and the dsDNA human adenovirus (HAdV) were determined in stored urine, sludge and manure, and were compared to results from controlled laboratory studies. The phages were chosen to span a range of susceptibilities to solution conditions typical of HEAM, and to represent different genome types.^16^ HAdV was chosen as a representative of a base-resistant human virus. Finally, experiments were conducted to determine the effect of HEAM-associated environmental parameters, such as microbial activity or the presence of metals, on inactivation.

## 2 Materials and methods

### 2.1 Virus and cells

HAdV type 2 was provided by Rosina Gironès (Laboratory of Virus Contaminants of Water and Food, University of Barcelona). HAdV was propagated on A549 human lung carcinoma epithelial cells, provided by the University Hospital of Lausanne. A549 cells were cultivated in high-glucose, pyruvate Dulbecco's modified Eagle's medium (DMEM; Invitrogen). The media was supplemented with penicillin (20 U mL^−1^), streptomycin (20 µg mL^− 1^) (Invitrogen), and 2 or 10% fetal bovine serum (FBS; Invitrogen) and cells were incubated at 37 °C in 5% CO_2_ and 95% humidity. Viruses were propagated by spiking 10 µl of HAdV (10^10^–10^11^ most probable number of cytopathogenic units (MPNCU) mL^−1^) into 160 cm^2^ flasks (TPP Techno Plastic Products, Trasadingen, Switzerland) containing 95% confluent cells, and were purified as described in Bosshard *et al.*^17^ From each flask, 1 ml of samples containing 10^10^–10^11^ MPNCU mL^−1^ of HAdV was collected and stored at 4 °C as virus stocks for the experiments. New stocks were produced before each set of experiments. Virus titers were determined as MPNCU mL^−1^ from 5 × 100 µl of samples on 96-wells plates (Greiner Bio-One, Frickenhausen, Germany) as described in Bosshard *et al.*^17^ Briefly, the DMEM containing 10% FBS on a 95% confluent cell monolayer was replaced by 100 µl of virus solution and augmented with 200 µl of DMEM containing 2% FBS. Cytopathogenic units could be discerned after an incubation time of 14 days. The detection limit was 10^2^–10^3^ MPNCU mL^−1^.

### 2.2 Phages and bacteria

Coliphages MS2 (DSMZ 13767) and ΦX174 (DSMZ 4497) and their host *Escherichia coli* (DSMZ 5695 and DSMZ 13127, respectively) were purchased from the German Collection of Microorganisms and Cell Cultures (DSMZ, Braunschweig, Germany). Coliphage T4 and its *E. coli* host (B1) were provided by Petr Leiman (Laboratory of Structural Biology and Biophysics, EPFL). Media used to grow *E. coli* B1 were free of any antibiotics. All phages were propagated and purified as described previously.^18^ Stock solutions were stored in the fridge, and the same stocks were used for all the experiments. Infectivity was assessed using the double agar layer method as described elsewhere.^19^ The detection limit for all phages was 300 PFU mL^−1^.

### 2.3 Stored urine, sludge and manure

Undiluted stored human urine was obtained from the Swiss Federal Institute of Aquatic Sciences (Eawag) in Dübendorf, Switzerland (CH), where urine is collected from waterless urinals and men's NoMix toilets and women's NoMix toilets respectively. Three batches of male stored urine taken in 3 different years (2012, 2013, 2014) and one batch of female stored urine (2014) were obtained and used for experimentation. An additional batch of stored urine was collected from urine diverting dry toilets in Durban, South Africa (SA) in 2014. Diluted stored urine was obtained by mixing undiluted stored urine (male, CH, 2012) and MilliQ water at urine: water ratios of 1 : 1, 1 : 2 and 1 : 9. In total, the various stored urine batches and dilutions yielded 15 urine samples (U1–U15; Table 1). For experiments with sludge, three batches of sludge were used: two batches (S1, S3) consisted of a synthetic fecal sludge. This sludge was made of walnuts, straw flour, kaolinite, sodium phosphate, ammonium chloride and potassium nitrate, and was digested by an inoculum of bacteria obtained from a thermophilic anaerobic digester, as described by Gallandat *et al.*^20^ An additional batch (S2) consisted of fecal sludge collected from septic tanks in Switzerland. Two batches of pig (M1, M2) and cow manure (M3, M4) were collected in the Swiss country-side.

**Table 1:** Main physical–chemical characteristic of stored human urine, sludge and manure. Concentrations ofmajor ions are listed in the supplementary material

ID	Sample description			*T* [°C]	pH	EC [mS cm^−1^]	Dilution^a^	Total solids%	{NH_3_} [mmol L^−1^]	Virus tested
U1	Urine	CH	Male (2012)	20	8.47		1:0		15.8	MS2
U2					8.49		1:1		9.6	MS2
U3					8.45		1:9		1.9	MS2
U4				35	8.15		1:0		19.1	MS2, HAdV
U5					8.19		1:0		24.6	MS2
U6					8.19		1:0		24.4	MS2
U7					8.22		1:1		13.6	MS2
U8					8.15		1:1		12.2	MS2
U9					8.19		1:2		8.4	MS2
U10					8.13		1:9		2.5	MS2
U11					8.13		1:9		2.7	MS2
U12		CH	Male (2013)	35	8.72	33.6	1:0		81.0	MS2, HAdV, ϕX174, T4
U13		CH	Male (2014)	35	8.79	33.0	1:0		106.0	MS2
U14		CH	Female	35	8.49	16.0	1 : 0		28.2	MS2
U15		SA	Mix	35	8.48	33.6	1:0		71.1	MS2
S1	Sludge	CH	Synthetic	35	8.24			3.3	27.7	MS2
S2		CH	Septic tank	35	7.76			6.7	1.3	MS2, HAdV, ϕX174, T4
S3		CH	Synthetic	35	7.39			2.5	0.2	MS2
M1	Manure	CH	Pig	35	7.79			2.3	8.4	MS2
M2		CH		35	8.08			2.0	8.5	MS2
M3		CH	Cow	35	8.05			4.6	35.7	MS2
M4		CH		35	8.23			1.7	14.2	MS2, HAdV, ϕX174, T4

a Urine : water ratio.

Upon arrival in the lab, the stored urine, sludge and manure were stored at 4 °C from weeks to months until use. Prior to characterization, all urine samples were centrifuged at 10 000 × *g* for 10 minutes. For sludge and manure characterization, 5–10 ml of MilliQ water were added, and the sample was shaken for 10–15 minutes and centrifuged at 4000 × *g* for 15 minutes. Stored urine, sludge and manure physical and chemical characteristics (summarized in Tables 1 and S1†) were determined as follows: pH was measured at experimental temperature (780 pH Meter with primatrode with NTC no. 6.0228.010, Metrohm, Herisau, Switzerland); the total ammonium nitrogen (TAN; NH_4_^+^/NH_3_) concentration was determined by ion chromatography (ICS-3000, IonPacCS16 column) with electrical conductivity detection (Dionex, Switzerland); phosphate, sulfate and chlorine concentrations were measured by ion chromatography (ICS-3000, IonPac AS11-HCcolumn); magnesium, calcium, potassium and sodium by inductively coupled plasma optical emission spectrometry (ICP-OES, Ciros, Spectro Analytical Instruments, Kleve, Germany); soluble chemical oxygen demand (SCOD) with cuvette tests (Hach-Lange, Berlin, Germany) and total inorganic carbon (TIC) by means of a TOC-TN Analyser (IL 550, Hach-Lange, Berlin, Germany). For TAN measurements, samples were diluted in 0.01 M HCl and for TIC measurements in 0.01 M NaOH to avoid loss of NH_3_ and CO_2_, respectively. For all other ions, samples were diluted in MilliQ water. Additionally, total solids (TS) were determined in sludge and manure according to standard methods.^21^ Given the low TS (<7%), one Liter of sludge or manure was considered as one kg. This characterization was generally performed in quadruplicate for stored urine and in triplicate for sludge and manure (see below for details). For male stored urine (2012) (U1–U11, see Table 1), the ion content was determined in the undiluted sample (U1)only, and was extrapolated for the rest of the samples composed of this urine (U2–U11). Only pH and TAN were determined under each experimental condition (U1–U11; see Table S1†).

Ion activities in each matrix were determined as a function of experimental temperature, pH and solution composition using PHREEQC (version 2.18.00)^22^ and a database using the Pitzer approach for calculating ion activities.^23^ The concentrations expressed in mol kg^−1^ in PHREEQC were considered equivalent to mol L^−1^. SCOD was transformed to acetate equivalents, because acetate was shown to represent 47% of the SCOD in stored urine.^24^ The transformation was determined according to the following stoichiometric relation:

C^2^H^3^O^2^ + 1.75O^2^ → 2CO^2^ + 1.5H^2^O

Manure was additionally tested for the presence of somatic and F-RNA coliphages. Only somatic coliphages were detected in pig manure at >10^3^ PFU g^−1^.

### 2.4 Experimental setup

ΦX174 and T4 were tested in one batch of stored urine, one batch of sludge and one batch of manure; HAdV in two batches of stored urine, one batch in sludge and one batch of manure; and MS2 in all the batches described in Table 1. An overview over the experimental setup is provided in the supplementary material (Scheme S1†). Prior to each inactivation experiment, the matrices were stored at the targeted experimental temperature during one (urine) or five to ten (manure, sludge) days to establish a stable temperature and microbial activity as well as to avoid drastic changes in solution composition during the inactivation phase.

**Stored urine experiments.**For experiments in stored urine, one milliliter of a MS2, ΦX174, T4 or HAdV solution containing 10^7^–10^10^ PFU or MPNCU mL−1 in virus dilution buffer (VDB; 5 mmol L^−1^ NaH_2_PO_4_, 10 mmol L^−1^ NaCl, pH 7.5) was added to airtight 116 ml glass serum flasks (Infochroma) containing 114 ml of stored urine solution. One mL samples were periodically withdrawn from each flask with a sterile syringe and were filtered through a 0.22 µm filter (Millipore).

**Sludge and manure experiments.** For sludge and manure, 1 mL of a MS2, ΦX174, T4 or HAdV solution (10^7^–10^10^ PFU or MPNCU mL^−1^ in VDB) was added to 150–300 ml of sludge or manure. The suspension was stirred during 5 minutes in a 500 mL closed bottle and 12 mL aliquots were then transferred into 15 ml falcon tubes (Sarstedt, Nümbrecht), which were tightly closed with a cap and parafilm. Two grams of samples were periodically collected from sacrificial falcon tubes, and were weighed and mixed with 10 ml beef extractsolution (BES; 100 g L^−1^ beef extract (Merck), pH 7.2) to promote the elution of viruses from solids. Samples were then shaken for 10–15 minutes and centrifuged for 20 minutes at 4000 × *g* at room temperature.^25^ Preliminary experiments yielded a 95% recoveryof MS2 spiked in sludge with this procedure. 1 ml of the supernatant was then withdrawn with a sterile syringe and filtered through a 0.22 µm filter (Millipore).

**Buffer experiment.** HAdV inactivation was further assessed in phosphate carbonate buffer (50 mmol L^−1^ carbonate and 40 mmol L^−1^ phosphate) at pH 9.0 and 35 °C as described previously.^15^

**Enumeration.** The filtered urine, sludge or manure samples were directly diluted in medium containing 2% FBS (HAdV) or VDB (MS2, ΦX174, T4), and were stored at 4 °C for no more than six hours prior to enumeration. Each stored urine was tested in duplicate flasks and sludge and manure were tested in triplicate tubes for all organisms. Phage titers were determined in duplicate or triplicate from the same reactor, whereas HAdV was enumerated once perreactor.

**Testing the role of microbiological activity and cations.** The effect of HEAM-associated parameters on inactivation was tested in stored urine and manure. Urine-specific components were investigated in samples of 1 : 9 diluted stored urine (U10) with MS2. To test the role of microbial activity, MS2 was exposed to samples filtered at 0.22 µm to remove live microorganisms. To inhibit enzyme activity, samples were first filtered at 0.22 µm to remove microorganisms and the remaining enzymes were heat-inactivated at 65 °C for 30 minutes. To minimize the influenceof cations on inactivation, the complexing agent ethylenediaminetetraacetic acid (EDTA; Acros) was added to stored urineto obtain a final concentration of 10 and 50 mmol L^−1^. The role of microbial and enzyme activity in manure (M4) was tested by exposing MS2 to heat-sterilized manure (65 °C for 1 h).

### 2.5 Data analysis

Inactivation kinetics were determined by least-square fit of the data to a first-order model:

$$C(t)=C_{0}{\text{e}}^{\text{-}k_{obs}t}$$( 1 )

where *C*_0_ and *C* [PFU or MPNCU mL^−1^] are the virus concentrations at time 0 (initial) and *t*, and *k*_obs_ is the first-order inactivation rate constant [day^−1^]. Non-first-order inactivation kinetics were fitted to a biphasic model:

$$C(t)=C_{0,\text{fast}}{\text{e}}^{\text{-}k_{obs,\text{fast}}t}+C_{0,\text{slow}}{\text{e}}^{\text{-}k_{obs,\text{slow}}t}$$( 2 )

where *C*_0,fast_ and *C*_0,slow_ indicate the initial concentrations, and *k*_obs,fast_ and *k*_obs,slow_ the inactivation rate constants for the fast- and slow-inactivating populations, respectively.

### 2.6 Model to predict MS2 inactivation

MS2 inactivation rate constants were determined in all stored urine, sludge, and manure samples, over a wide range of solution conditions and experimental temperatures. This data set was used to challenge the predictive model of MS2 inactivation discussed in Decrey *et al*.,^15^ which was established for well-controlled solutions. The model demonstrated that MS2 infectivity loss can be related to the degradation (transesterification) of the viral genome by different bases. Specifically, the inactivation rate constants of MS2 in laboratory solutions could be predicted as follows:

$$k_{\text{pred}}=\sum_{\text{j}}\{j\}\cdot k_{j}$$( 3 )

where *k*_pred_ is the predicted first-order rate constant [day^−1^], {j} is the activity in [mol L^−1^] of bases (or nucleophiles) present in solutions that participate in the base-catalyzed transesterification of ssRNA, and *k*_j_ [day^−1^ L mol^−1^] is the second order inactivation rate constant associated with the inactivating species j. *k*_j_ can be determined according to the Brønsted catalysis law:

$$\log_{10}k_{j}=\beta .{\text{p}k}_{a}+D$$( 4 )

where the parameter *β* is the Brønsted coefficient, and *D* is a constant, both specific to MS2. For MS2 inactivation, we observed *β* = 0.41 at 35 °C. To predict inactivation at different temperatures, the temperature-dependence of each parameter in the Brønsted catalysis law (eqn (4)) must be considered. However, literature reports on a similar reaction, namely the base-catalyzed decomposition of nitramide, showed no relevant temperature dependence of the Brønsted plot slopes for a temperature range from 15–45 °C.^26,27^ Correspondingly, in our studies, the coefficient *β* determined experimentally at 20 °C and 35 °C were equal (see ESI,† Fig. S1). We therefore assumed a constant value of *β* over the temperature range considered.

In contrast to *β*, p*K*_a_, *k*_j_ and *D* are dependent on temperature. For any given temperature, *D*(*T*) was estimated based on available experimental data^15^ for j = NH_3_ asfollows:

$$D(T)=\text{l0}{\text{g}}_{10}k_{\text{N}{\text{H}}_{3}}(T)-0.41.\text{p}K_{{\text{aNH}}_{3}}(T)$$( 5 )

All p*K*_a_(*T*) values were calculated according to eqn (S1) (see ESI,† determination of p*K*_a_), and *k*_NH3_(*T*) was determined by the Arrhenius relationship reported in Decrey *et al.*:^15^as follows

$$k_{\text{N}{\text{H}}_{3}}=\exp\left(-5356.5\frac{1}{T}+22.043\right)$$( 6 )

Finally, it is apparent that only those bases contribute to inactivation of MS2 that are present at a significant activity (eqn (3)), and that have a conjugated acid with a relatively high p*K*_a_ (eqn (4)). Giventhe composition of the matrices used herein (Tables 1 and S1†), the only bases (j) considered were therefore OH^−^, NH_3_, CO_3_^2−^, HCO_3_^−^, PO_4_^3−^ and HPO_4_^2−^. Note that a user-friendly interface for the predictive model is available for free online (https://lodecrey.shinyapps.io/MS2inactivation/).^28^

## 3 Results and discussion

### 3.1 Inactivation kinetics

**Fig. 1:**
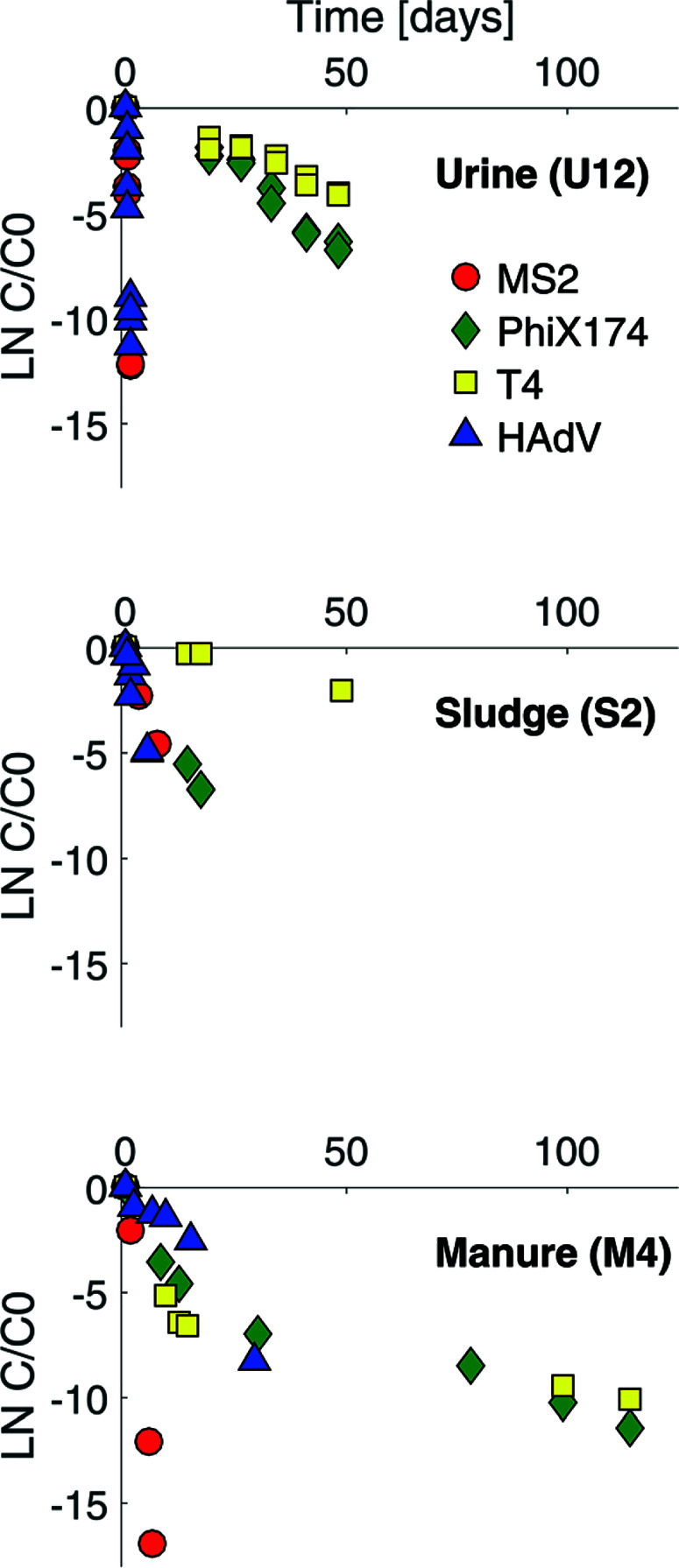
Inactivation curves for MS2 (red circles), ΦX174 (green diamonds), T4 (yellow squares) and HAdV (blue triangles) in stored urine (U12), sludge (S2) and manure (M4). The complete set of inactivation curves obtained in this study are shown in the ESI† (Fig. S2 and S3).

Example virus inactivation curves obtained in one batch of human urine, sludge and manure are shown in Fig. 1 (see ESI,† Fig. S2 and S3 for the complete set of inactivation curves). Viruses in all matrices studied exhibited a loss of infectivity with time, and inactivation followed first-order kinetics, except in the case of ΦX174 and T4 in manure (Fig. 1 and S3†). First-order inactivation rate constants and coefficients of determination are listed in Table S2 (ESI†). First-order inactivation kinetics were also reported by Höglund *et al*.^29^ and Vinnerås *et al.*^30^ for Salmonella phage 28B and rotavirus in stored urine. In contrast to the findings herein, however, Vinnerås *et al.*^30^ observed a two phase inactivation behavior for MS2 and ΦX174, with an initial fast inactivation followed by a slow first-order reduction.

The first-order inactivation kinetics observed herein indicate that solutions conditions were stable over the course of the inactivation experiments. Correspondingly, a characterization of the composition of stored urine over time showedthat both pH and ion concentrations were stable when stored at 20 °C or 35 °C. For sludge and manure, however, changes in solution conditions were observed (ESI,† Table S3). For example, in the case of sludge (S2), the concentration of TAN doubled over the course of 14 days, and the concentrations of Ca^2+^, Mg^2+^, SO_4_^2−^ decreased more than two-fold whereas the pH and other ions were stable; in the case of manure (M1), the pH increased from 7.58 to 8.0 and TIC doubled whereas other ions were stable. The effect of the changing solution conditions on inactivation, however, remained small: predicted inactivation rate constants of MS2 (see section 2.6) differed by less than a factor of 1.4 for most matrices over the duration of an experiment (see ESI,† Table S3). Consequently, for any downstream analysis, the average of the initial and final composition was used for S2, S3, M1, M2 and M3 (Tables 1 and S1†); for S1 and M4, only the composition at initial time was assessed.

### 3.2 Validation of MS2 inactivation prediction in stored urine, sludge and manure

**Fig. 2:**
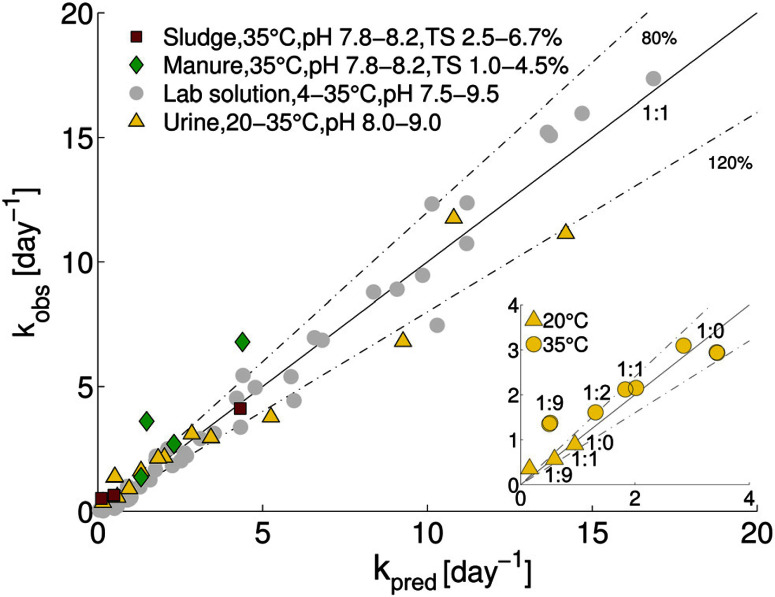
Comparison of measured and predicted MS2 inactivation rate constants for stored urine, sludge and manure. For comparison, data from lab solutions (Decrey *et al*.;^15^ grey circles) are also shown. Values of *k*_pred_ were determined from eqn (3) and (4). The solid line represents a 1 : 1 relation between measurement and prediction (*k*_pred_/*k*_obs_ = 1). Dashed lines indicate 80% and 120% of *k*_pred_/*k*_obs_ (*i.e.*, *k*_pred_/*k*_obs_ = 0.8 and 1.2 respectively). The inset shows *k*_pred_ *versus k*_obs_ for different dilutions of urine. The urine : water ratio for each data point is indicated.

The comparison between predicted and observed inactivationrate constants is shown in Fig. 2. Over half of the predictions fell within 80–120% of *k*_obs,_ and three-fourths of the predictions were within 60–140% of *k*_obs_. This ability of the model to accurately predict MS2 inactivation supports the notion that the dominant underlying mechanism of inactivation is basecatalyzed transesterification of the ssRNA genome.^15,16^ This appears to be the case in both synthetic solutions and in real matrices such as stored urine, and sludge and manure. Consistent with our results, Gao *et al.*^31^ recently reported that during mesophilic anaerobic treatment of Coxsackievirus, the viral genome was the main inactivating target.

**Fig. 3:**
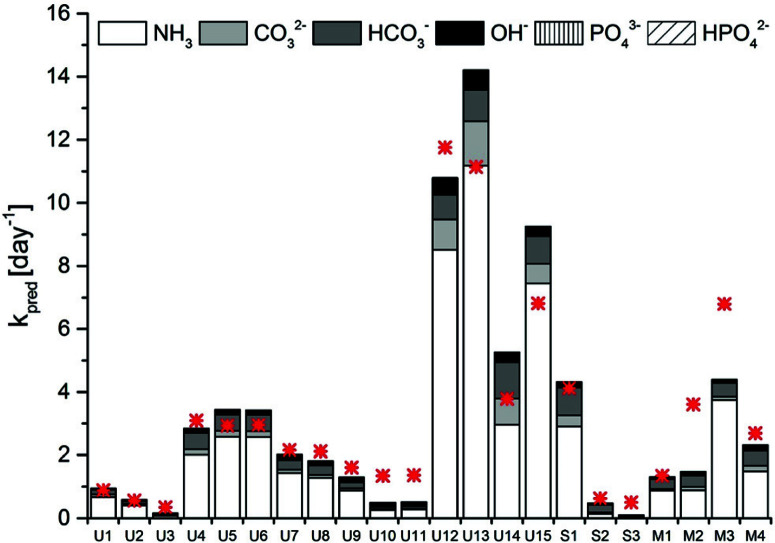
Contribution of the main bases present in stored urine, sludge and manure to the *k*_pred_ of MS2. The measured *k*_obs_ is depicted by the red asterisk.

A model sensitivity analysis was conducted to assess the influence of pH and temperature, and the inclusion of measured ion concentrations in the model, on the accuracy of the prediction (Fig. S4†). Specifically, we re-assessed the model prediction for all 22 samples at either pH values of 0.1 units surrounding the measured value or at temperaturesof 1 °C surrounding the measured temperature. In addition, predictions were carried out that included only TAN, or TIC and TAN, but none of the other ions in solution. This analysis revealed similar sensitivity to shifts resulting from changes in pH by 0.1 units and from changes in temperature by 1 °C (Fig. S4 and Table S2†). A relatively minor error in the measurement may thus lead to an inaccurate *k*_pred_. Interestingly, no relevant differences were observed if all ions were taken into account in the prediction, as compared to only the TIC and TAN (Fig. S4 and Table S2†). Thus, measurements of temperature, pH, TIC and TAN are sufficient to obtain a reasonably accurate prediction of MS2 inactivation rate constants. Removal of TIC from the prediction led to a lower accuracy(Fig. S4 and Table S2†). This highlights the importance of carbonate in MS2 inactivation in HEAM. Carbonate and bicarbonate contributed between 15 and 40% to the total *k*_obs_ in stored urine, sludge and manure (Fig. 3). The contribution of carbonate species was even higher (>50%) in sludge S2, which had a pH < 8.0 and equivalent amounts of TIC and TAN.

### 3.3 Influence of HEAM-associated parameters on MS2 inactivation

Despite the quite high accuracy of the predicted inactivation rate constants for MS2 in all matrices considered, some small deviations from the measured *k*_obs_ were observed: for some stored urine samples, the model yielded a slight overestimate of the rate constant compared to the observation, while the model tended to underestimate inactivation in 1 : 9 diluted urine, sludge and manure (Fig. 2). These deviations may be in part linked to imprecise measurements of the ion concentrations and the PHREEQC estimation of the ion activities. Alternatively, underestimations could indicate that additional inactivating processes occur that are not accounted for in the model. In this context, we assessed the role of two HEAM-associated parameters in inactivation, namely biological (microbial or enzymatic) activity and metal ions, and we discussed the influence of additional bases not considered by our model.

#### 3.3.1 Microbiological activity

**Fig. 4:**
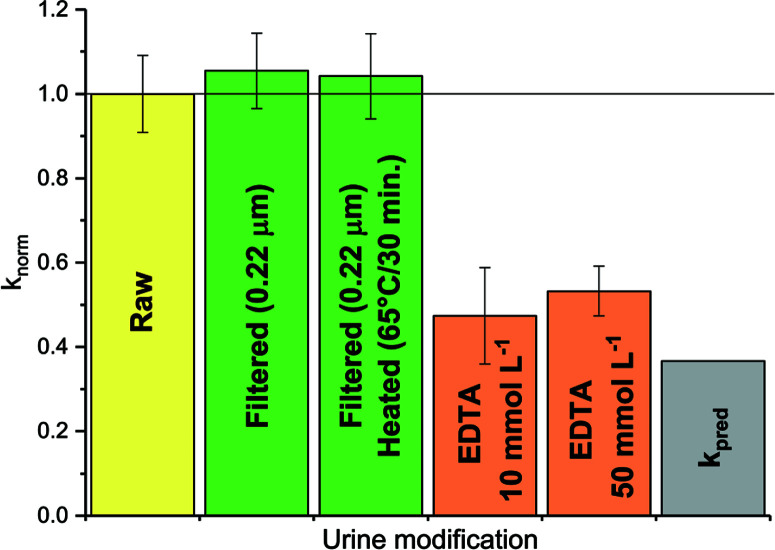
MS2 inactivation rate constants determined at 35 °C in 1:9 diluted, raw or modified urine. Modifications were filtration and heating to remove microbial or enzymatic activity (green), and addition of EDTA to complex metal ions (orange). Also shown are the rate constants in raw urine *k*_obs_ (yellow) and the predicted rate constant *k*_pred_ (grey). *k*_norm_ corresponds to the ratio of the *k*_obs_ determined in the modified urine samples and *k*_obs_ in raw 1 : 9 diluted urine (yellow). Error bars depict 95% confidence interval associated with *k*_norm_.

Stored urine is known to possess bactericidal properties,^12,13,32^ which may limit the effects of microbiological activity on viruses in undiluted urine. Upon urine dilution, however, the concentration of bactericidal substances diminishes, such that a more active microbial population may be established. Consequently, the effect of microbiological activity on virus survival could be greater in diluted than in undiluted urine. Such a scenario would account for the under-predicted inactivation rate constant in diluted urine (Fig. 2). However, neither filtration to remove microorganisms, nor heating of the diluted urine to inactivate enzymes, affected the observed MS2 inactivation kinetics (Fig. 4, green bars). Hence, microbiological activity could not account for the differences between *k*_obs_ and *k*_pred_ in 1 : 9 diluted urine. Heat-sterilization of manure (M4) even led to an increase in inactivation, resulting in a *k*_obs_ of 4.6 (±0.7) day^−1^ (data not shown) compared to 2.7 (±0.8) day^−1^ in untreated manure. Thus, MS2 seemed insensitive to biological activity. Similar results were observed in soil saturated with secondary effluent by Nasser *et al.*,^33^ and in nitrifying urine by Bischel *et al.*^34^ In contrast, Mondal *et al.*^35^ reported that MS2 could be inactivated by both commercial and sludge-derived protease solutions. At sufficiently high protease concentrations, biological activity may thus compete with chemicallymediated MS2 inactivation in HEAM, though this situation was not encountered herein.

#### 3.3.2 Metal ions

Metal ions are known to accelerate base-catalyzed RNA transesterification,^36,37^ and may thus enhance MS2 inactivation in HEAM compared to laboratory solutions. This transesterification-accelerating mechanism is currently not considered in *k*_pred_. To reduce the effect of free metal ions, 1 : 9 diluted urine was amended with EDTA, a metal complexing agent. This was found to decrease *k*_obs_ to a value close to *k*_pred_ (see Fig. 4, orange bars). Thus, the presence of EDTA suppressed the action of the urine constituents responsible for the higher *k*_obs_. Interestingly, if ammonia (as NH_4_Cl) was added to (EDTA-free) 1 : 9 diluted urine, *k*_obs_ increased as expected based on the increase in {NH_3_} (see ESI,† Fig. S5). The effect of ammonia was thus additive to that of the metal ions, resulting in an overall higher inactivation than in other urine matrices with the same NH3 activity. We currently cannot explain, however, why urine dilution, followed by ammonia addition results in higher inactivation than an equivalent ammonia activity in undiluted urine.

The role of metal ions was not explicitly studied in sludge and manure. Nevertheless, literature reports indicate that particularly manure contains metal cations up to the mmol L^−1^ concentration range.^38,39^ It is thus reasonable to conclude that metal ions in these matrices also contribute to MS2 inactivation.

#### 3.3.3 Other bases

Finally, underestimation of the true inactivation may result from the limited range of bases considered (OH^−^, NH_3_, CO_3_^2−^, HCO_3_^−^, PO_4_^3−^ and HPO_4_^2−^). Odorous compounds such as sulfide (p*K*_a1,2_ = 6.99, 12.92) and 4-methylphenol (p*K*_a_ = 10.6), which have high p*K*_a_ values may also contribute to the base-catalyzed inactivation. The concentration of total sulfide and 4-methylphenol were shown to be in the mmol L^−1^ range in stored urine and fecal sludge.^40,41^ The effect of these bases on inactivation remains to be tested.

### 3.4 Influence of genome type and microbial activity on virus inactivation kinetics in urine, sludge and manure

As discussed above, considerable heterogeneity exists among the inactivation kinetics of viruses with different genome types. Specifically, in well-controlled solutions, DNA viruses were shown to be more persistent than ssRNA viruses. To validate this trend in real matrices, we compared the inactivation of ssRNA MS2 phage to that of three DNA viruses, specifically phage ΦX174 (ssDNA), phage T4 (dsDNA) and HAdV (dsDNA). Inactivation kinetics were assessed in a subset of stored urine, sludge and manure samples at 35 °C, and were compared to those obtained in laboratory solution with and without NH_3_.^15,16^

As expected, the ssRNA virus MS2 was inactivated more readily than the DNA viruses in most matrices, (Fig. 1 and ESI† Table S2). This confirmed the higher sensitivity of ssRNA viruses to NH3 and mildly alkaline pH. In undiluted stored urine at 35 °C (U12), a four log10 (99.99%) inactivation was achieved within one day for MS2, whereas it took more than 100 days to reach the same level of inactivation for T4. In sludge and manure the differences among ssRNA and DNA viruses were smaller, with a four log_10_ loss being achieved within 15 and 207 days in sludge (S2) and 3.5 and 40 days in manure (M4) at 35 °C for MS2 and T4 respectively. A possible reason for the narrower range of inactivation kinetics among ssRNA and DNA viruses in sludge and manure is the contribution of microbial or enzymatic activity to inactivation. While microbiological inactivation is not relevant for MS2 compared to its rapid chemical inactivation (see section 3.3.1), it may accelerate the inactivation of DNA viruses beyond the slow chemical inactivation kinetics in HEAM, in particular in matrices with high microbiological activity.

**Fig. 5:**
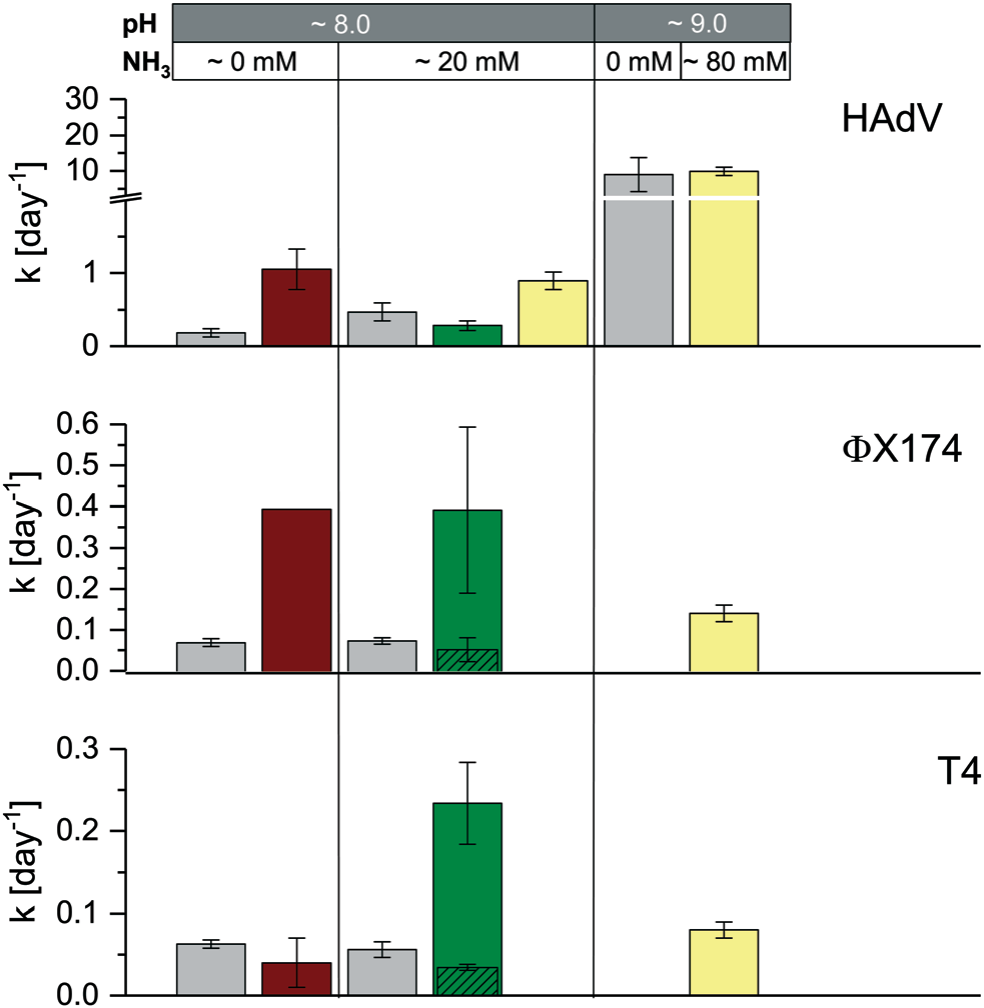
Comparison of virus inactivation in laboratory solution (grey bars), stored urine (U4 [pH 8.0, 20 mmol L^−1^ NH_3_], U12 [pH 9.0, 80 mmol L^−1^ NH_3_]), sludge (S2) and manure (M4) at 35 °C. Kinetics in laboratory solution (from left ot right: phosphate carbonate buffer [pH 8.0, 50 mmol L^−1^ carbonate, 60 mmol L^−1^ phosphate] and ammonium carbonate [pH 8.0, 50 mmol L^−1^ carbonate, 20 mmol L^−1^ NH_3_]) were derived from Decrey *et al.*,^16^ except for HAdV at pH 9.0 (phosphate buffer [pH 9.0, 50 mmol L^−1^ carbonate, 40 mmol L^−1^ phosphate]). The exact rate constants for U4, U12, S2 and M4 are listed in Table S2.† For ΦX174 and T4 in M4, columns with diagonal patterns depict *k*_obs_slow_. Error bars depict 95% confidence interval associated with *k*_obs_.

Consistent with our previous findings, HAdV was the most readily inactivated among the DNA viruses, and it was more resistant than MS2 in stored urine (U4) at pH 8.2 and approximately 20 mmol L^−1^ NH_3_ and in manure (M4) (Fig. 1 and ESI† Table S2). However, in stored urine with a higher pH and NH3 content (U12) and in sludge (S2), the inactivation of HAdV increased, resulting in similar to greater inactivation rate constants compared to MS2 (Fig. 1 and ESI† Table S2). A comparison with controlled solutions of similar physical– chemical properties revealed that pH could account for the fast inactivation observed in U12 (Fig. 5). In contrast, inactivation in S2 could not be explained by the known physical– chemical parameters, indicating a potential contribution of microbiological processes.

Faster than expected inactivation was also observed for ΦX174 and T4 in manure. Specifically, these two phages exhibited biphasic inactivation behavior in manure with an initial fast inactivation followed by a secondary, slower phase (eqn (2); ESI,† Fig. S6). For both phages, *k*_obs_fast_ was 4–5 times larger than *k*_obs_slow_ (Fig. 5, green bars) and the *k*_obs_slow_ generally corresponded to the *k*_obs_ in a controlled solution with the same physical–chemical properties (Fig. 5, grey bars). The first, rapid decrease in infective virus thus is caused by parameters associated with manure that are not present in laboratory solutions, whereas the second, slower phase may be attributed to the effect of solution conditions only. We propose that the biphasic inactivation kinetics is associated with virus adsorption onto the manure. Specifically, while irreversible adsorption can be ruled out as a removal mechanism (given the high recovery of infective virus in our experimental protocol; see Materials and methods), adsorption to solids may lead to the protection of viruses from microbial and enzymatic activity. The fast, initial inactivation could thus be dominated by microbiological inactivation of suspended viruses, whereas the slower phase results from physical–chemical inactivation of viruses adsorbed to solids. This hypothesis is consistent with the findings by others that demonstrate faster inactivation in the liquid- than in the solid-associated virus fraction during anaerobic digestion^42^ and in wastewater.^43^

Microbiological contributions to virus inactivation have previously been reported for HAdV, Hepatitis A virus, norovirus and enteroviruses in a range of matrix types.^33,44–51^ In most cases, inactivation could only be partly attributed to biological processes, whereas physical–chemical matrix components also played a role. Unfortunately, the matrix composition was usually poorly characterized, and it is therefore difficult to parameterize its influence in inactivation. It appears, however, that the most important matrix components and their resulting virucidal activities vary widely depending on both the matrix and virus type. For example, it was shown that septic tank effluent digestion was more efficient at inactivating viruses when mixed with dairy cattle or swine manure slurry.^50,51^ The same authors observed that among 31 bacterial strains isolated from animal manure, only 10 proved to be efficient at inactivating virus. Here, we propose inactivating microbial or enzymatic activity in sludge but not in manure for HAdV, in manure but not in sludge for T4, and in both for ΦX174 (Fig. 5). The distinct matrix and virus parameters that result in inactivation, however, remain to be determined.

The complex nature of virus inactivation in real matrices is further reflected in the literature, where a large variation in virus inactivation kinetics in excreta is reported. Consequently, results contradicting our data can be found: for example, others have shown ΦX174 to be inactivated as fast as MS2 in stored urine^12^ and stored fecal sludge,^14^ and HAdV to be inactivated faster than MS2 in fecal sludge.^52^ Similarly, ssRNA phage f2 and coxsackievirus exhibited comparable inactivation to rotavirus (dsRNA) during mesophilic anaerobic digestion of sludge with, albeit at low pH (~7.3).^53^ On the other hand, other studies provided observations consistent with our data. For example, F-RNA specific coliphages were shown to be more sensitive than somatic (DNA) coliphage and dsDNA Salmonella phage 28B during mesophilic digestion of raw sewage sludge^54^ and the organic fraction of municipal solid waste.^55^ Furthermore, somatic coliphages were found to exhibit low sensitivity to the addition of urea, calcium carbonate and sodium percarbonate used to sanitize composted sewage sludge, which is consistent with our finding that ΦX174 and T4 are not affected by the main chemical components of stored urine and sludge.^56^ Overall, the influence of solids content on the inactivation of different virus types remains poorly understood and needs to be further elucidated. In particular, more research is needed on matrices with lower liquid fractions, such as fecal sludge (solids content between 20–95% (ref. 14)).

## 4 Conclusions

Among enteric viruses, the vast majority have an ssRNA genome. A good understanding of the factors that promote the inactivation of ssRNA viruses during waste treatment is therefore particularly important. The data presented herein demonstrate that for ssRNA phage MS2, inactivation kinetics and mechanisms established in laboratory solutions are transferable to real matrices. We furthermore established that carbonate, ammonia, pH and temperature are important drivers of inactivation that need to be maximized to enhance treatment efficiency. Future studies should be extended to additional ssRNA viruses to more conclusively establish the optimal treatment conditions.

While viruses with other genome types are not as common, some enteric viruses with public health relevance have DNA and dsRNA genomes (*e.g*., adenovirus, polyomavirus, rotavirus). For DNA and dsRNA viruses, kinetic insights developed in controlled solutions may not be as readily transferable to real matrices, due to the potential contribution of microbiologically driven inactivation processes in HEAM. The kinetics based on physical–chemical parameters established in laboratory solutions can therefore only be considered as worst case scenarios for the inactivation of DNA and dsRNA viruses in HEAM.

Discrepancies exist between our results and literature reports, as well as among different literature reports. To reconcile those, a better understanding of the mechanisms involved in virus inactivation in HEAM is needed. While we believe that we have a good handle on main mechanism involved in the inactivation of ssRNA viruses, those responsible for DNA and dsRNA virus inactivation remain to be determined. In this context, we again emphasize the potential contribution of microorganisms on virus inactivation. Determining the virus properties that render it susceptible to microbiological inactivation will be an important next step in understanding inactivation in HEAM.

Finally, this study helps identify appropriate proxies to monitor virus inactivation in HEAM. Specifically, our data confirms that somatic coliphage such T4 or ΦX174 are conservative indicators of resistant ssDNA, dsDNA and dsRNA viruses. However, they are too stable to serve as indicators for the inactivation of the more labile HAdV. The utility of these indicators as suitable proxies for further DNA or dsRNA viruses should thus be confirmed.

## References

[1] Albihn A., Nyberg K., Ottoson J. and Vinneras B., *Sustain. Agric. Ecosyst. Heal. Sustain. Agric. B*. 1, 2012.

[2] WHO, *Guidelines for the safe use of wastewater, excreta and greywater - v. 4. Excreta and greywater use in agriculture*, World Health Organisation, 2006.

[3] Emmoth E., *Virus inactivation – evaluation of processes used in biowaste management, PhD thesis, Diss*. Swedish Univ *Agric. Sci*., Uppsala, Sweden, 2010, ISBN 978-91-576-9001-2.

[4] Pell A. N., *J. Dairy Sci*., 1997, 80, 2673–2681. 9361239 10.3168/jds.S0022-0302(97)76227-1 PMC7130904

[5] Strande L., Ronteltap M. and Brdjanovic D., *Faecal Sludge Management — Systems Approach Implementation and Operation*, IWA Publishing London, 2014.

[6] Lim S. S., et al, *Lancet*, 2012, 380, 2224–2260. 23245609 10.1016/S0140-6736(12)61766-8 PMC4156511

[7] Mara D., Lane J., Scott B. and Trouba D., *PLoS Med*., 2010, 7(11), e1000363. 21125018 10.1371/journal.pmed.1000363 PMC2981586

[8] Ngure F. M., Reid B. M., Humphrey J.H., Mbuya M. N., Pelto G. and Stoltzfus R. J., *Ann. N. Y. Acad. Sci*., 2014, 1308, 118–128. 24571214 10.1111/nyas.12330

[9] Bartram J. and Cairncross S., *PLoS Med*., 2010, 7(11), e1000367. 21085694 10.1371/journal.pmed.1000367 PMC2976722

[10] Hawkins P., Blackett I. and Heymans C., The missing link in sanitation service delivery: a review of fecal sludge management in 12 cities, *Water and Sanitation Program*, 2014.

[11] Tilley E., Ulrich L., Lüthi C., Reymond P. and Zurbrügg C., Compendium of Sanitation Systems and Technologies (2nd Revised Edition), Swiss Fed. *Inst. Aquat. Sci*. Technol. (Eawag), Dübendorf, Switzerland, 2014, ISBN: 978-3-906484-57-0.

[12] Vinneras B., Nordin A., Niwagaba C. and Nyberg K., *Water Res*., 2008, 42, 4067–4074. 18718625 10.1016/j.watres.2008.06.014

[13] Fidjeland J., Magri M. E., Jönsson H., Albihn A. and Vinnerås B., *Water Res*., 2013, 47, 6014–6023. 23941983 10.1016/j.watres.2013.07.024

[14] Magri M. E., Philippi L. S. and Vinnerås B., *Appl. Environ. Microbiol*., 2013, 79, 2156–2163. 23335764 10.1128/AEM.03920-12 PMC3623211

[15] Decrey L., Kazama S., Udert K. M. and Kohn T., *Environ. Sci. Technol*., 2015, 49, 1060–1067. 25496714 10.1021/es5044529

[16] Decrey L., Kazama S. and Kohn T., *Appl. Environ. Microbiol*., 2016, 82, 4909–4920. 27260358 10.1128/AEM.01106-16 PMC4968548

[17] Bosshard F., Armand F., Hamelin R. and Kohn T., *Appl. Environ. Microbiol*., 2013, 79, 1325–1332. 23241978 10.1128/AEM.03457-12 PMC3568621

[18] Pecson B. M., Martin L. V. and Kohn T., *Appl. Environ. Microbiol*., 2009, 75, 5544–5554. 19592538 10.1128/AEM.00425-09 PMC2737914

[19] Kohn T. and Nelson K. L., *Environ. Sci. Technol*., 2007, 41, 192–197. 17265947 10.1021/es061716i

[20] Gallandat K., Evaluation of pathogens inactivation during thermophilic anaerobic digestion of faecal sludge, *Msc thesis, EPF Lausanne*, 2014.

[21] APHA, AWWA and WEF, *Standard Methods for the Examination of Water and Wastewater*, 1999.

[22] Parkhurst D. L. and Appelo C. A. J., *Model. Tech*. B. 6, 2013, p. 497.

[23] Hafner S. D. Bisogni J. J., *Water Res*., 2009, 43, 4105–4114. 19664794 10.1016/j.watres.2009.05.044

[24] Larsen T. A., Udert K. M. and Lienert J., *Source Separation and Decentralization for Wastewater Management*, IWA Publishing, 2013.

[25] Guzmán C., Jofre J., Blanch A. R. and Lucena F., *J. Virol. Methods*, 2007, 144, 41–48. 17499367 10.1016/j.jviromet.2007.03.017

[26] Baughan E. C. and Bell R. P., *Proc. R. Soc. London, Ser. A*, 1937, 158, 464–478.

[27] Liotta S. and La Mer V. K., *J. Am. Chem. Soc*., 1938, 60, 1967–1974.

[28] Decrey L., Available on: https://lodecrey.shinyapps.io/MS2inactivation/, 2015.

[29] Höglund C.Ashbolt N. , Stenstrom T. A. and Svensson L., *Adv. Environ. Res*., 2002, 6, 265–275.

[30] Vinnerås B., Nordin A., Niwagaba C. and Nyberg K., *Water Res*., 2008, 42, 4067–4074. 18718625 10.1016/j.watres.2008.06.014

[31] Gao T.Tong Y. Cao M.Li X. and Pang X., *Water Res*., 2013, 47, 4259–4264. 23764576 10.1016/j.watres.2013.04.046

[32] Chandran A., Pradhan S. K. and Heinonen-Tanski H., *J. Appl. Microbiol*., 2009, 107, 1651–1657. 19457041 10.1111/j.1365-2672.2009.04353.x

[33] Nasser A. M., Glozman R. and Nitzan Y., *Water Res*., 2002, 36, 2589–2595. 12153026 10.1016/s0043-1354(01)00461-4

[34] Bischel H. N., Schertenleib A., Fumasoli A., Udert K. M. and Kohn T., *Environ. Sci.: Water Res. Technol*., 2015, 1, 65–76.

[35] Mondal T., Rouch D. A., Thurbon N., Smith S. R. and Deighton M. A., *J. Water Health*, 2015, 13, 459–472. 26042978 10.2166/wh.2014.313

[36] Forconi M. and Herschlag D., *Methods Enzymol*., 2009, 468, 91–106. 20946766 10.1016/S0076-6879(09)68005-8

[37] Corona-Martínez D. O., Gomez-Tagle P. and Yatsimirsky A. K., *J. Org. Chem*., 2012, 77, 9110–9119. 22991967 10.1021/jo301649u

[38] Nicholson F. A., Chambers B. J., Williams J. R. and Unwin R. J., *Bioresour. Technol*., 1999, 70, 23–31.

[39] McBride M. B. and Spiers G., *Commun. Soil Sci. Plant Anal*., 2001, 32, 139–156.

[40] Marti B., Udert K. M. and Morgenroth E., Bioelectrochemical Removal of Sulfur from Source-Separated Urine, *Msc Thesis, ETH Zurich*, 2010, p. 69.

[41] Lin J., Aoll J., Niclass Y., Velazco M. I., Wünsche L., Pika J. and Starkenmann C., *Environ. Sci. Technol*., 2013, 47, 7876–7882. 23829328 10.1021/es401677q

[42] Sanders D. A., Malina J. F., Moore B. E., Sagik B. P. and Sorber C. A., *J. - Water Pollut. Control Fed.*, 1979, 51, 333–343. 225573

[43] Ye Y., Ellenberg R. M., Graham K. E. and Wigginton K. R., *Environ. Sci. Technol*., 2016, 50, 5077–5085. 27111122 10.1021/acs.est.6b00876

[44] Ward R. L., *Appl. Environ. Microbiol*., 1982, 43, 1221–1224. 6285823 10.1128/aem.43.5.1221-1224.1982 PMC244213

[45] Cliver D. O. and Herrmann J. E., *Water Res*., 1972, 6, 797–805.

[46] Knowlton D. R. and Ward R. L., *Appl. Environ. Microbiol*., 1987, 53, 621–626. 3034153 10.1128/aem.53.4.621-626.1987 PMC203725

[47] Carratalà A., Rusiñol M., Rodriguez-Manzano J., Guerrero-Latorre L., Sommer R. and Girones R., *Food Environ. Virol*., 2013, 5, 203–214. 10.1007/s12560-013-9123-3 23955425

[48] Chaudhry R. M., Nelson K. L. and Drewes J. E., *Environ. Sci. Technol*., 2015, 49, 2815–2822. 25642587 10.1021/es505332n

[49] Deng M. Y. and Cliver D. O., *Microb. Ecol*., 1995, 30, 43–54. 24185411 10.1007/BF00184512

[50] Deng M. Y. and Cliver D. O., *Appl. Environ. Microbiol*., 1995, 61, 87–91. 7887630 10.1128/aem.61.1.87-91.1995 PMC167263

[51] Deng M. Y. and Cliver D. O., *Appl. Environ. Microbiol*., 1992, 58, 2016–2021. 1320368 10.1128/aem.58.6.2016-2021.1992 PMC195720

[52] Magri M. E., Fidjeland J., Jönsson H., Albihn A. and Vinnerås B., *Sci. Total Environ*., 2015, 520, 213–221. 25817758 10.1016/j.scitotenv.2015.03.035

[53] Spillmann S. K., Traub F., Schwyzer M. and Wyler R., *Appl. Environ. Microbiol*., 1987, 53, 2077–2081. 2823708 10.1128/aem.53.9.2077-2081.1987 PMC204061

[54] Astals S., Venegas C., Peces M., Jofre J., Lucena F. and Mata-Alvarez J., *Water Res*., 2012, 46, 6218–6227. 23063441 10.1016/j.watres.2012.07.035

[55] Ottoson J. R., Schnurer A. and Vinneras B., *Lett. Appl. Microbiol*., 2008, 46, 325–330. 18266645 10.1111/j.1472-765X.2007.02317.x

[56] Häfner F., *Optimised Ammonia Sanitisation of Sewage Sludge*, *Msc thesis*, SLU Uppsala, 2014.

