# Supplementary material for: Virus inactivation in stored human urine, sludge and animal manure under typical conditions of storage or mesophilic anaerobic digestion
Source: Environ Sci (Camb). 2017 Mar 17;3(3):492–501. doi: 10.1039/c6ew00311g (PMC7705124; doi:10.1039/c6ew00311g)
Supplement: Supplementary file 1 [file ESWRT-03-492-s001.pdf]

Electronic Supplementary Material (ESI) for Environmental Science: Water Research & Technology.
This journal is © The Royal Society of Chemistry 2017

Supplementary material
for

Virus inactivation in stored human urine, sludge and animal manure under typical conditions of storage or mesophilic anaerobic digestion

Loïc Decrey[a] and Tamar Kohn[a]*

[a]*Laboratory of Environmental Chemistry, School of Architecture, Civil and Environmental Engineering (ENAC), École Polytechnique Fédérale de Lausanne (EPFL), CH-1015 Lausanne, Switzerland*

*Corresponding author
phone: +41 (0)21 693 0891; fax: +41 (0)21 693 8070;
e-mail: tamar.kohn@epfl.ch

Determination of pKa (as a function of T)

- For all species (except phosphate) (according to PHREEQC empirical pK_a determination)

$$log_{10}K_a = A_1 + A_2T + \frac{A_3}{T} + A_4 log_{10}T + \frac{A_5}{T^2} \quad \text{(S1)}$$

With:

	A1	A2	A3	A4	A5
CO3 2-	107.8975	0.032528	-5151.79	-38.9256	563713.9
HCO3 -	356.3094	0.06092	-21834.4	-126.834	1684915
NH3	0.6322	-0.00123	-2835.76	0	0
Kw	-283.971	-0.0507	13323	102.2445	-1119669

And for OH^- (K_a = [OH][H]/[H_2O] = K_w/55.5): $pKa\ (T) = -log_{10}\left(\frac{K_{w\ (T)}}{55.5}\right)$

- For phosphate (Van't Hoff equation):

$$pK_{a_2} = -log_{10}\left(10^{-pKa_1} \exp\left(\frac{-\Delta H}{R}\left(\frac{1}{T_2} - \frac{1}{T_1}\right)\right)\right) \quad \text{(S2)}$$

With:

ΔH_r (standard enthalpy of reaction) at 25°C:

PO4/HPO4	-14.7 kJ/mol[1]
HPO4/H2PO4	-4.2 kJ/mol

And $\Delta H_r = \Delta H_f$ (product)- ΔH_f (reactant) (from online handbook of chemistry and physics):

PO4	-1277.4 kJ/mol
HPO4	-1292.1 kJ/mol
H2PO4	-1296.3 kJ/mol

[1] Confirmed by PHREEQC database (phreeqc.dat) -3.53 kcal/mol = -14.7 kJ/mol

Scheme S1: Illustration of experimental protocol

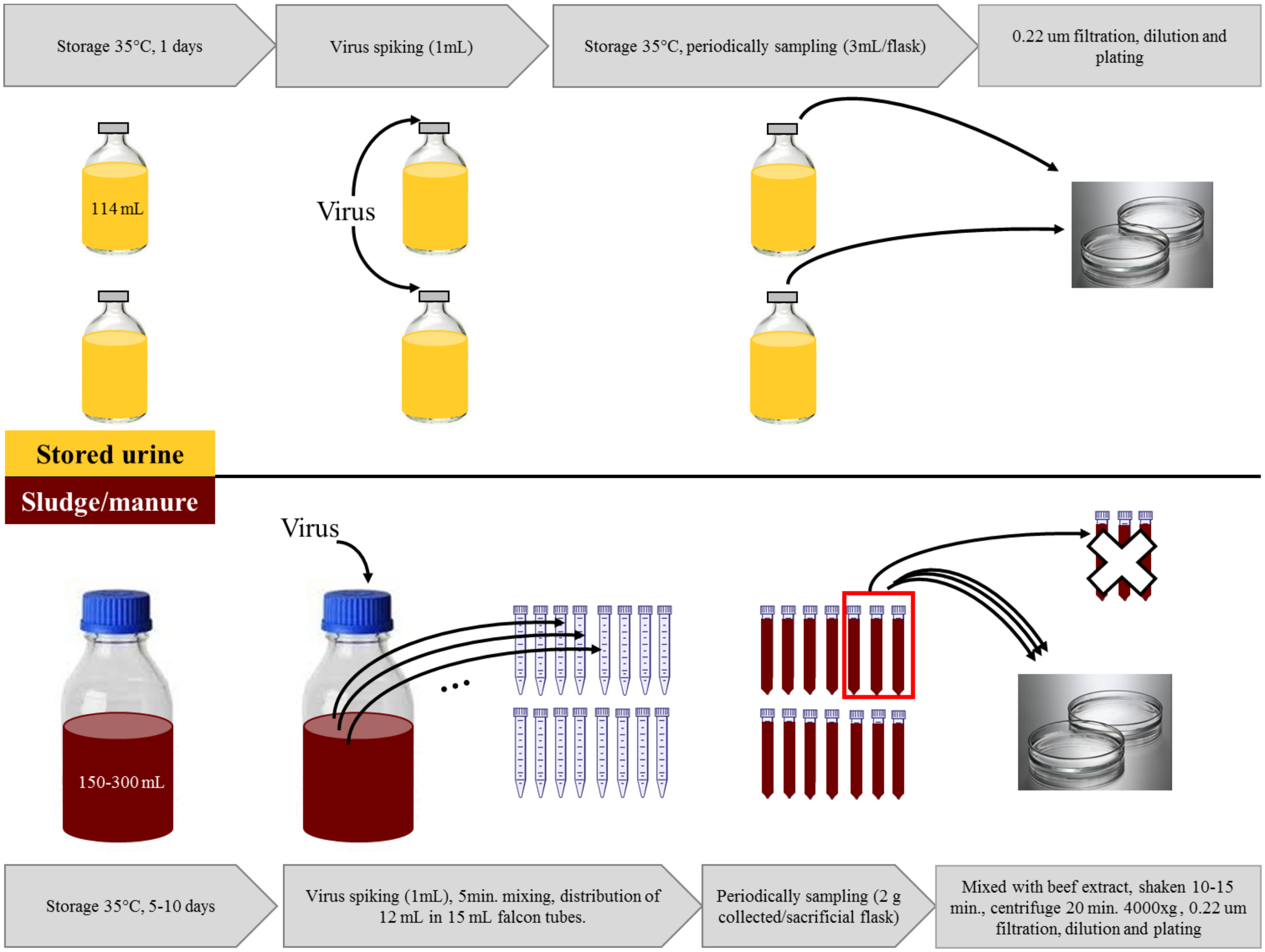

Table S1: Composition of matrixes used in this study. For those matrices analyzed at the beginning and end of an experiment, the average values are listed. 95% CI indicates the 95% confidence interval of three (sludge and manure) or four (urine) replicate measurements. SCOD = soluble chemical oxygen demand; TAN = total ammonium nitrogen; TIC = total inorganic carbon.

ID	Sample description			Ca		Cl		SCOD		K		Mg		Na		TAN		PO_4		TIC		SO_4	
				[mM]	95% CI	[mM]	95% CI	[mgO_2/L]	95% CI	[mM]	95% CI	[mM]	95% CI	[mM]	95% CI	[mM]	95% CI	[mM]	95% CI	[mM]	95% CI	[mM]	95% CI
U1	Urine	CH	male (2012)	0.51	0.06	98.2	5.6	5063	278	34.0	1.3	0.05	0.01	84.3	2.8	231.8	23.8	6.33	0.38	109.7	3.7	7.9	0.71
U2				0.25		49.1		2531		17.0		0.03		42.2		123.2	18.1	3.16		54.8		3.9	
U3				0.05		9.8		506		3.4		0.01		8.4		22.7	0.6	0.63		11.0		0.8	
U4				0.51	0.06	98.2	5.6	5063	278	34.0	1.3	0.05	0.01	84.3	2.8	213.9	12.2	6.33	0.38	109.7	3.7	7.9	0.71
U5				0.51	0.06	98.2	5.6	5063	278	34.0	1.3	0.05	0.01	84.3	2.8	254.0	26.3	6.33	0.38	109.7	3.7	7.9	0.71
U6				0.51	0.06	98.2	5.6	5063	278	34.0	1.3	0.05	0.01	84.3	2.8	252.3	21.6	6.33	0.38	109.7	3.7	7.9	0.71
U7				0.25		49.1		2531		17.0		0.03		42.2		119.6	23.0	3.16		54.8		3.9	
U8				0.25		49.1		2531		17.0		0.03		42.2		124.4	3.8	3.16		54.8		3.9	
U9				0.15		29.5		1519		10.2		0.02		25.3		74.3	11.5	1.90		32.9		2.4	
U10				0.05		9.8		506		3.4		0.01		8.4		22.5	2.4	0.63		11.0		0.8	
U11				0.05		9.8		506		3.4		0.01		8.4		23.9	0.1	0.63		11.0		0.8	
U12		CH	male (2013)	0.19	0.02	98.7	4.1	4085	54	32.9	0.8	<0.01		69.2	1.9	319.7	10.9	10.84	0.43	208.6	1.1	7.9	0.24
U13		CH	male (2014)	0.18	0.01	89.8	3.1	3635	212	30.0	1.3	<0.01		63.6	1.8	378.2	18.4	0.78	0.02	283.4	68.9	8.2	0.22
U14		CH	female	0.25	0.01	37.1	19.3	367	64	13.4	0.6	0.02	0.00	24.8	0.8	160.0	19.3	0.28	0.01	263.8	154.1	3.5	0.17
U15		SA	mix	0.21	0.02	118.7	3.3	2004	50	21.1	0.6	0.05	0.06	99.9	1.8	434.7	9.8	11.67	1.85	213.6	7.9	10.6	0.20
S1	Sludge	CH	Synthetic	1.06	0.95	4.8	3.3	2782	789	4.0	1.2	0.38	0.37	8.2	2.8	245.3	144.8	0.17	0.05	171.1	120.4	0.2	0.04
S2		CH	Septic tank	0.89	0.74	3.8	1.1	438	115	1.4	0.2	0.48	0.40	2.8	0.4	27.8	11.1	2.10	0.08	39.6	5.6	0.1	0.10
S3		CH	Synthetic	0.37	1.32	17.8	0.6	2191	1429	25.7	1.0	0.41	1.58	2.0	0.1	10.9	4.3	1.60	0.90	9.4	7.8	0.4	0.00
M1	Manure	CH	Pig	1.66	12.97	19.9	0.2	9816	5136	20.1	0.2	1.29	0.01	11.4	0.1	196.4	7.6	0.30	0.30	64.4	38.5	0.9	0.10
M2		CH		1.38	1.91	15.5	0.3	4527	2543	15.8	0.2	1.70	1.02	9.2	0.1	99.1	6.6	0.44	0.24	68.1	22.8	0.4	0.09
M3		CH	Cow	1.10	[a]	22.2	0.3	6394	425	39.9	1.6	4.15	[a]	10.5	0.4	505.3	6.0	0.23	0.19	96.1	5.4	0.8	0.10
M4		CH		2.18	10.68	17.3	0.1	3100	1196	30.3	3.0	3.42	2.32	8.5	0.9	121.5	7.7	0.56	0.44	84.8	16.1	0.0	

[a] Only one measurement

Table S2: Measured (k_{obs}) and predicted (k_{pred}) inactivation rate constants.

		k_{obs}			k_{pred}						
						T sensistivity		pH sensitivity		Ions measured	
						-1°C	+1°C	-0.1	+0.1	only TIC & NH4	Only NH4
Virus		[day^{-1}]	IC 95%	R^2	[day^{-1}]	[day^{-1}]	[day^{-1}]	[day^{-1}]	[day^{-1}]	[day^{-1}]	[day^{-1}]
MS2	U1	0.89	0.06	0.995	0.95	0.84	1.07	0.79	1.14	0.94	0.78
	U2	0.56	0.02	0.998	0.59	0.52	0.67	0.49	0.71	0.58	0.49
	U3	0.35	0.02	0.998	0.16	0.14	0.18	0.13	0.19	0.15	0.13
	U4	3.09	0.25	0.990	2.85	2.55	3.17	2.41	3.38	2.83	2.40
	U5	2.93	0.10	0.998	3.44	3.08	3.84	2.89	4.11	3.42	3.02
	U6	2.95	0.20	0.997	3.43	3.07	3.82	2.87	4.09	3.41	3.00
	U7	2.15	0.11	0.995	2.02	1.81	2.25	1.69	2.40	1.99	1.71
	U8	2.12	0.15	0.997	1.83	1.64	2.04	1.53	2.18	1.80	1.53
	U9	1.61	0.10	0.991	1.31	1.18	1.46	1.10	1.57	1.29	1.10
	U10	1.35	0.14	0.984	0.50	0.45	0.56	0.42	0.60	0.49	0.41
	U11	1.37	0.24	0.982	0.52	0.46	0.58	0.43	0.62	0.51	0.43
	U12	11.76	0.38	0.999	10.80	9.72	11.90	9.19	11.80	10.50	10.00
	U13	11.14	1.15	0.989	14.20	12.80	15.60	12.20	16.40	13.40	13.00
	U14	3.78	0.45	0.978	5.25	4.75	5.80	4.55	6.06	4.65	3.70
	U15	6.82	0.46	0.991	9.26	8.31	10.30	7.77	11.00	8.88	8.51
	S1	4.12	[a]		4.32	3.88	4.80	3.67	5.08	3.83	3.25
	S2	0.63	0.19	0.995	0.49	0.44	0.54	0.44	0.55	0.45	0.21
	S3	0.51	0.06	0.997	0.11	0.10	0.12	0.10	0.13	0.11	0.05
	M1	1.35	0.35	0.986	1.31	1.18	1.46	1.11	1.55	1.32	1.03
	M2	3.60	0.57	0.999	1.48	1.33	1.64	1.26	1.74	1.44	1.10
	M3	6.79	5.35	0.967	4.39	3.92	4.91	3.62	5.32	4.35	4.07
	M4	2.69	0.81	0.983	2.31	2.08	2.57	1.96	2.72	2.18	1.77
HAdV	U4	0.89	0.12	0.982							
	U12	9.89	1.14	0.973							
	S2	1.05	0.28	0.884							
	M4	0.28	0.07	0.939							
T4	U12	0.08	0.01	0.964							
	S2	0.04	0.03	0.941							
	M4	0.23	0.05	0.997	(fast)						
		0.03	0.00	0.999	(slow)						
PhiX174	U12	0.14	0.02	0.957							
	S2	0.39	0.00	1.000							
	M4	0.39	0.20	0.986	(fast)						
		0.05	0.03	0.941	(slow)						

[a] k_{obs} determined with two data points only

Table S3: Solution composition and predicted inactivation rate constants for sludge and manure at the beginning and end of the kinetic experiments.

Sample ID			Sludge								Manure											
			S2				S3				M1				M2				M3			
			initial		final		initial		final		initial		final		initial		final		initial		final	
			0 day		14 days		0 day		21 days		0 day		10 days		0 day		4 days		0 day		2 days	
			Avg.	std	Avg.	std	Avg.	std	Avg.	std	Avg.	std	Avg.	std	Avg.	std	Avg.	std	Avg.	std	Avg.	std
Measured	TIC	[mM]	35.20	3.19	43.99	1.68	5.42	0.10	13.31	9.64	36.62	0.38	92.14	32.45	49.46	1.56	86.67	11.97	97.29	2.79	94.81	7.41
	TAN	[mM]	18.57	0.79	37.04	4.67	13.94	1.24	7.87	3.56	198.71	4.08	194.04	9.97	95.35	4.65	102.79	5.94	508.32	6.65	502.38	3.38
	Cl	[mM]	2.88	0.22	4.71	0.20	17.45	0.21	18.20	0.58	20.13	0.25	19.86	0.13	15.59	0.15	15.34	0.35	22.02	0.25	22.43	0.21
	SO4	[mM]	0.19	0.02	0.04	0.01	0.35	0.00	0.44	0.02	0.85	0.02	1.03	0.13	0.32	0.02	0.45	0.07	0.84	0.09	0.85	0.15
	PO4	[mM]	2.08	0.12	2.11	0.03	0.91	0.02	2.39	0.08	0.52	0.25	0.16	0.02	0.63	0.10	0.26	0.15	0.14	0.04	0.32	0.24
	Ca	[mM]	1.51	0.28	0.28	0.14	0.99	[a]	0.07	0.04	2.50	[a]	0.84	[a]	1.54	[a]	1.23	[a]	<0.01		1.11	[a]
	Mg	[mM]	0.81	0.15	0.15	0.08	1.15	[a]	0.04	0.03	1.29	[a]	1.29	[a]	1.62	[a]	1.78	[a]	<0.01		4.15	[a]
	Na	[mM]	2.47	0.06	3.15	0.05	2.01	0.02	1.89	0.00	11.44	0.10	11.35	0.12	9.14	0.14	9.17	0.09	10.39	0.43	10.75	0.04
	K	[mM]	1.22	0.01	1.60	0.01	26.58	0.04	24.78	0.12	20.18	0.12	20.07	0.27	15.80	0.23	15.71	0.20	39.29	1.78	40.81	0.31
	SCOD	[mgO2/L]	500.00	11.31	375.80	9.90	1404.15	28.36	2958.02	261.09	11658.86	1146.74	7973.81	4044.89	6735.84	163.22	2318.21	150.48	6560.65	319.16	6227.57	40.66
	pH	[-]	7.78	0.07	7.74	0.05	7.42	0.03	7.36	0.20	7.58	0.00	8.00	0.20	8.13	0.05	8.05	0.08	8.02	0.00	8.08	0.01
Calculated with PHREEQC	NH3	[mM]	0.94		1.70		0.32		0.148		5.29		13.1		8.86		8.38		33.8		37.8	
	CO3	[mM]	0.08		0.10		0.00544		0.0107		0.0439		0.299		0.223		0.348		0.293		0.324	
	HCO3	[mM]	25.70		32.00		3.79		8.53		21.1		54.8		30.3		56.9		51.2		49.3	
	OH	[mM]	0.00124		0.00113		0.000539		0.000469		0.000774		0.00204		0.00275		0.00229		0.00211		0.00243	
	PO4	[mM]	1.78E-05		1.62E-05		3.04E-06		6.89E-06		9.06E-07		8.19E-07		6.07E-06		2.22E-06		3.45E-07		8.65E-07	
	HPO4	[mM]	0.54		0.54		0.211		0.55		0.0436		0.015		0.0822		0.0362		0.00603		0.0132	
	k_{pred} [b]	$[day^{-1}]$	0.42		0.551		0.0988		0.126		0.79		2.05		1.41		1.6		4.18		4.62	

[a] Only one measurement

[b] Calculated with the model described in section 3.2

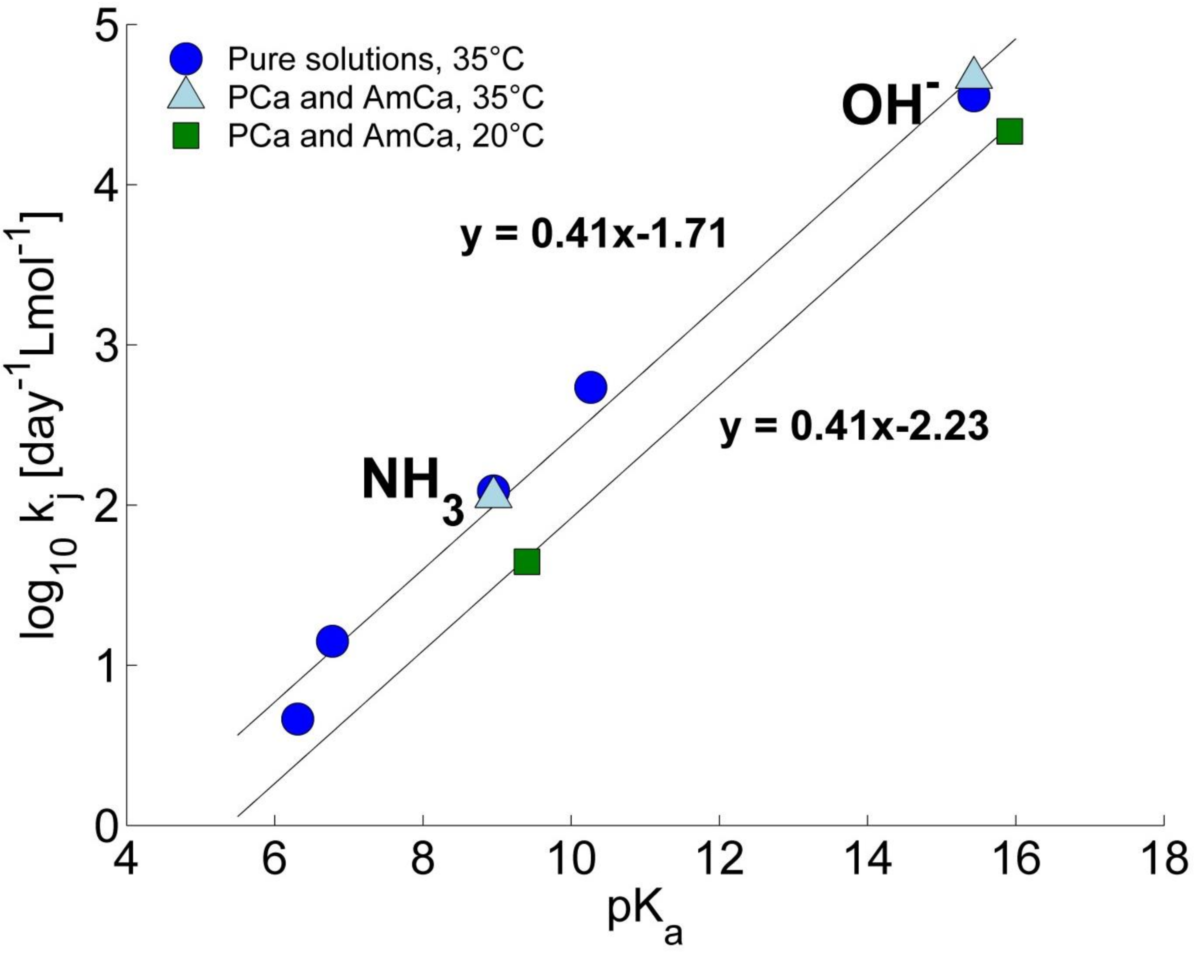

Figure S1: Bronsted plot of the pKa of various bases versus their second-order rate constant for inactivation of MS2. kj was determined, according to eq. 4, in aqueous solutions of the pure base solution at 35°C (circle) and in phosphate-carbonate (PCa) and ammonium-carbonate (AmCa) buffer solutions for bases OH and NH_3 respectively at 35°C (triangle) and 20°C (square). All data are derived from Decrey et al.[1]. pKa values were corrected for temperature (see above, determination of pKa).

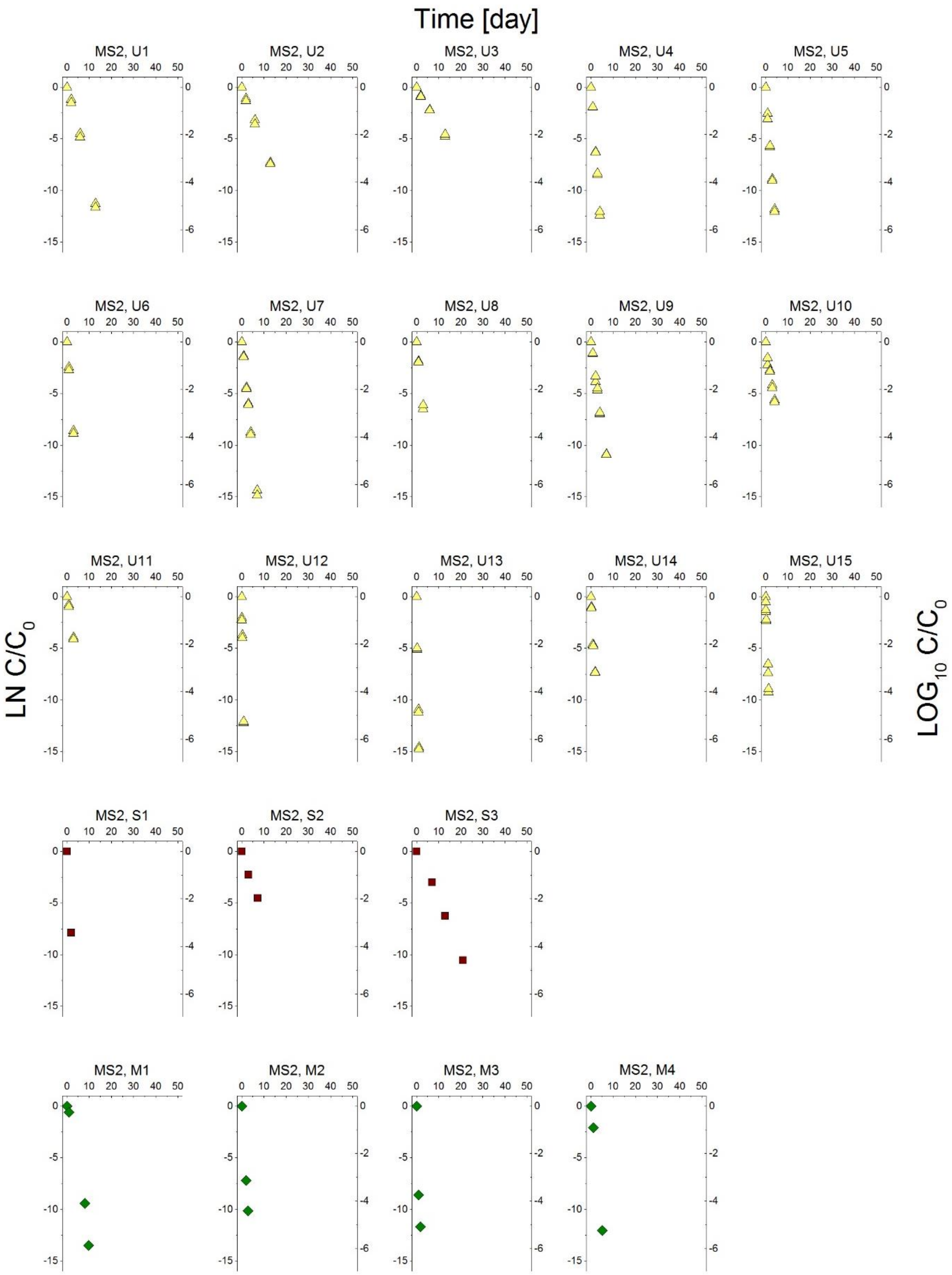

Figure S2: kinetics of MS2 inactivation in stored urine (U1-U15), sludge (S1-S3) and manure (M1-M4).

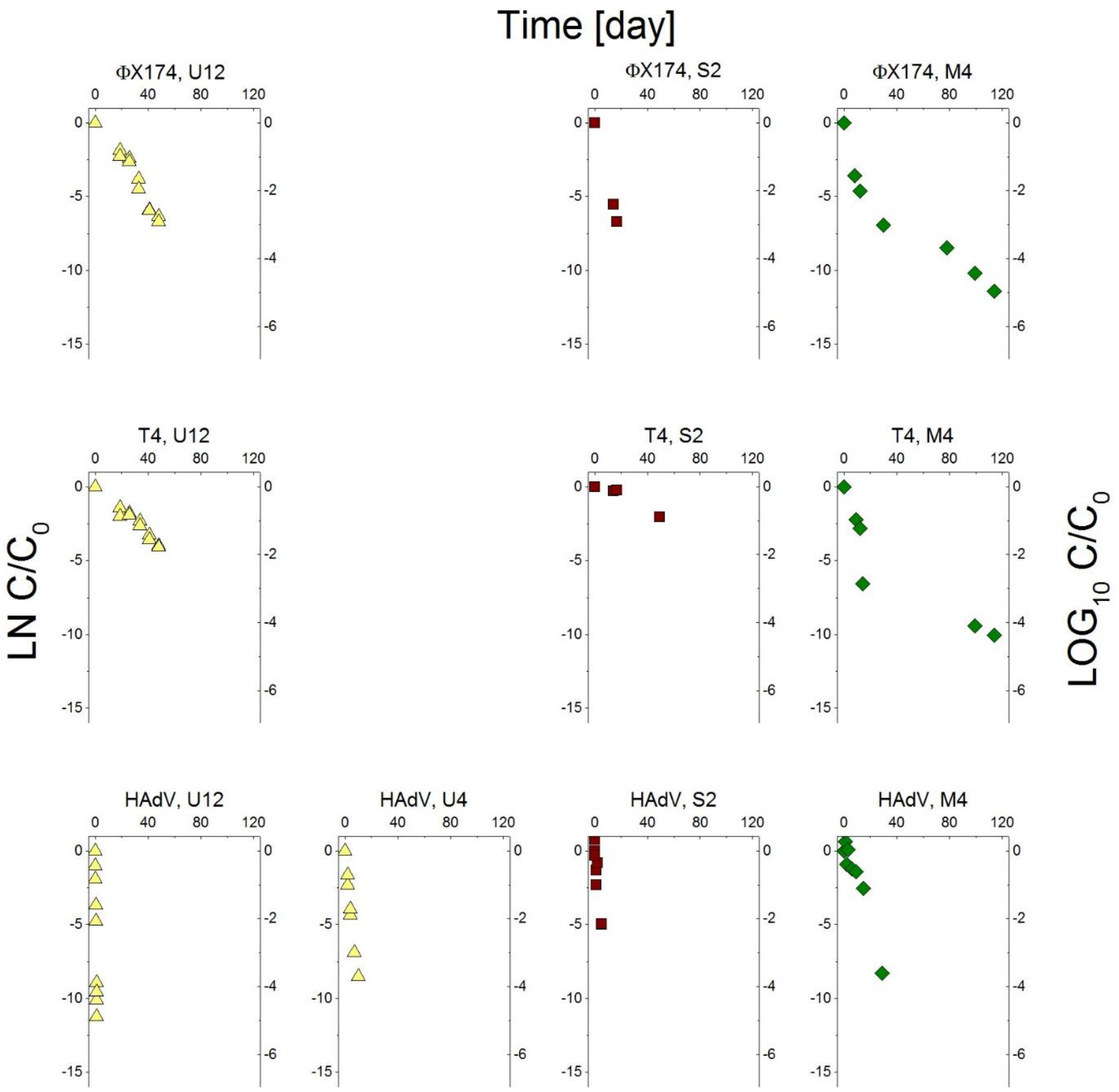

Figure S3: kinetics of ΦX174, T4 and HAdV inactivation in stored urine (U4, U12), sludge (S2) and manure (M4).

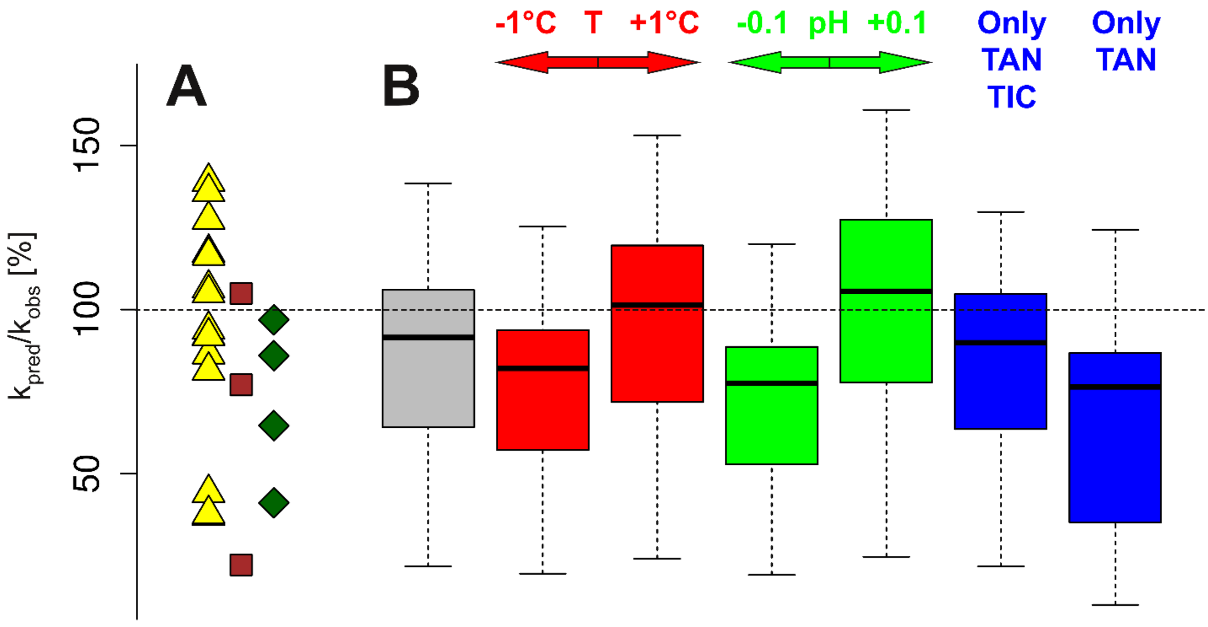

Figure S4: A. Accuracy of the predicted MS2 inactivation rate constant in stored urine (yellow triangles), sludge (brown squares) and manure (green diamonds). B. Sensitivity of the MS2 model prediction to temperature change (red), pH change (green) and TIC and TAN concentrations (blue) in stored urine, sludge and manure. The prediction corresponding to the measured properties of stored urine, sludge and manure (Tables 1 and S1) is represented in grey. Data are shown in boxplot format, where the thick line indicates the median, the box is bounded by the first and third quartiles (50% of the data) and the whiskers indicates the minimum and maximum of the ratio k_{pred}/k_{obs} (in percentage) determined for all 22 samples tested.

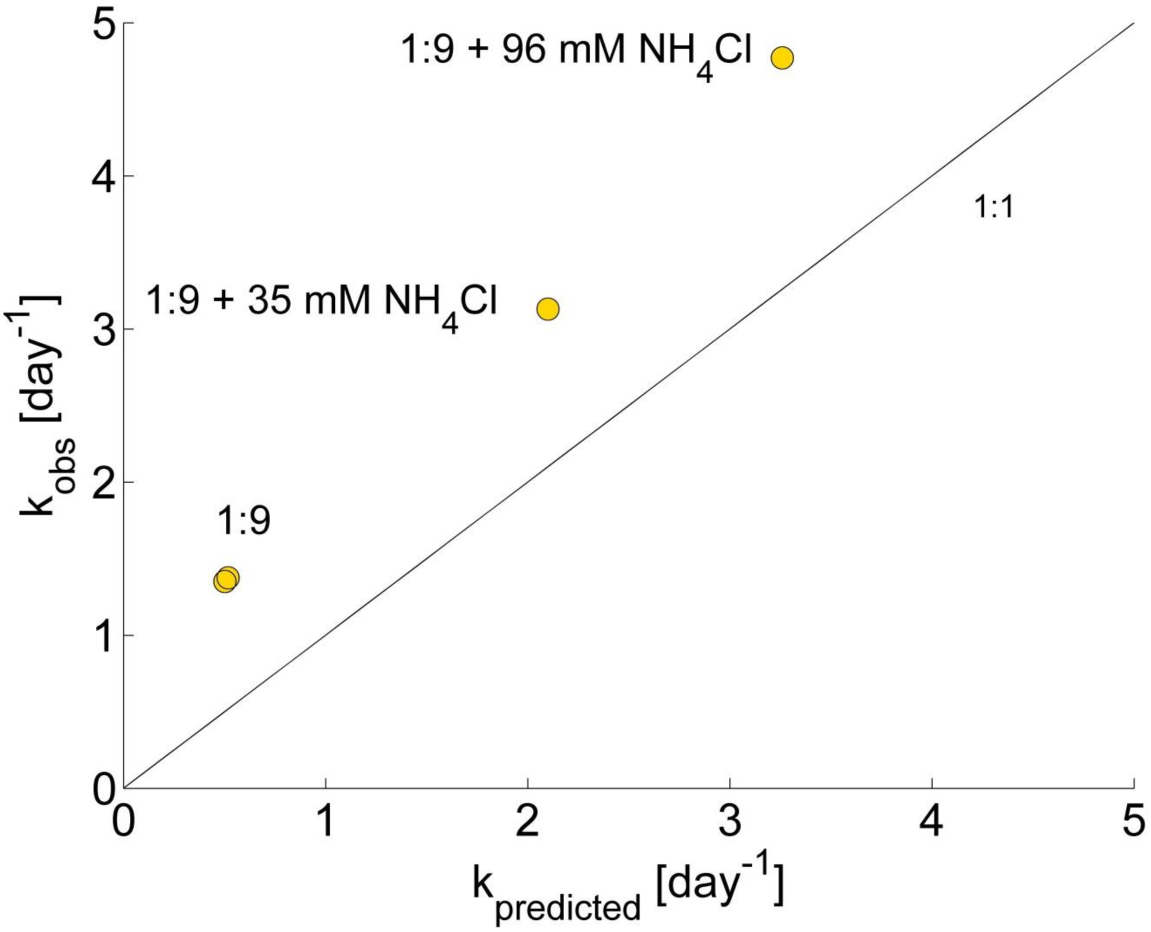

Figure S5: Comparison of measured and predicted inactivation rate constants for MS2 at 35°C in 1:9 diluted urine without NH_4Cl addition, and with 35 and 96 mmol L^{-1} NH_4Cl. NaOH (9.8 and 22.1 mmol L^{-1}) was added with NH_4Cl to partly compensate for pH drop. Final pH values are: 8.13, 8.52 and 8.46 from lowest to highest ammonia content. The solid line represents the 1:1 ratio between measurement and prediction.

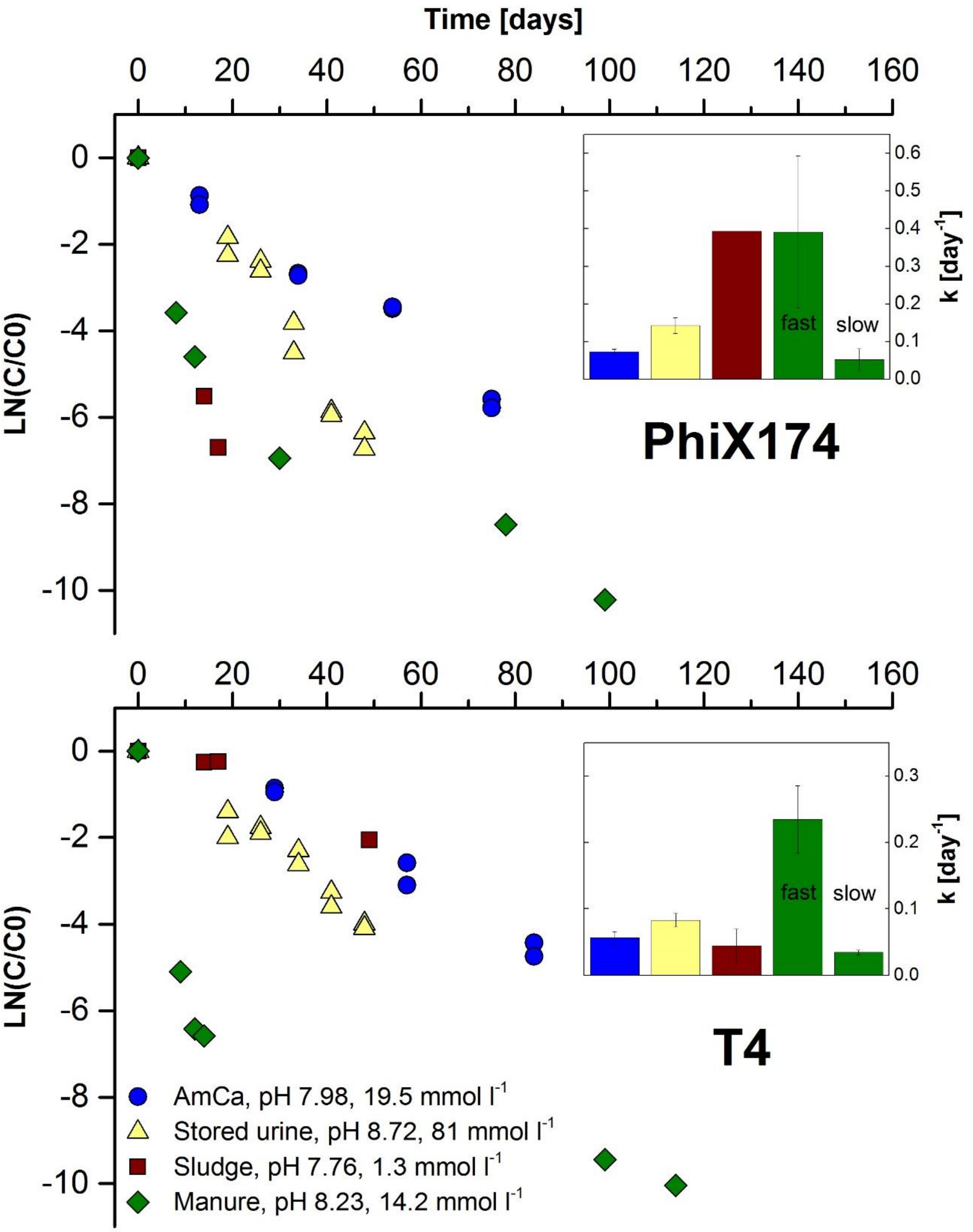

Figure S6: detailed kinetics of ΦX174 and T4 inactivation in buffer solution (data from Decrey et al.[2]), stored urine (U12), sludge (S2) and manure (M4). The insets summarize the inactivation rate constants for the initial fast and late slow phase of the inactivation curves in manure. pH and NH_3 activities of the different solutions are described in the legend.

References

1 L. Decrey, S. Kazama, K. M. Udert and T. Kohn, *Environ. Sci. Technol.*, 2015, **49**, 1060–1067.

2 L. Decrey, S. Kazama and T. Kohn, *Appl. Environ. Microbiol.*, 2016, **82**, 4909–20.
